# Oral management for a patient with trismus accompanied by Isaacs’ syndrome: a case report

**DOI:** 10.1186/s12903-024-04485-2

**Published:** 2024-06-22

**Authors:** Asuka Tani, Shinsuke Mizutani, Mitsuru Watanabe, Takashi Irie, Katsuhisa Masaki, Noriko Isobe, Haruhiko Kashiwazaki

**Affiliations:** 1 Section of Geriatric Dentistry and Perioperative Medicine in Dentistry, Division of Maxillofacial Diagnostic and Surgical Science, Faculty of Dental Science, Kyushu University, Fukuoka, Japan; 2 OBT Research Center, Faculty of Dental Science, Kyushu University, Fukuoka, Japan; 3 Department of Neurology, Neurological Institute, Graduate School of Medical Sciences, Kyushu University, Fukuoka, Japan

**Keywords:** Isaacs’ syndrome, Trismus, Neuromyotonia, Sleep bruxism, Case report

## Abstract

**Background:**

Isaacs’ syndrome, also known as neuromyotonia or peripheral nerve hyperexcitability, is a rare disorder that affects the peripheral nervous system. Clinical findings include cramps, fasciculations, and myokymia; however, there are few reports of dental treatment for trismus.

**Case presentation:**

A patient with trismus due to Isaacs’ syndrome experienced swelling and pain in the gingiva surrounding his right lower first molar. He was diagnosed with chronic apical periodontitis by a dentist near his home. However, the patient was informed that dental treatment and medication could not be administered because of the presence of Isaacs’ syndrome, and he visited the Geriatric Dentistry and Perioperative Oral Care Center at Kyushu University Hospital 2 weeks later. The patient’s painless mouth-opening distance (between incisors) was 20 mm at that time, and medication, including amoxicillin capsules and acetaminophen, was administered because the dental extraction forceps or endodontic instruments were difficult to insert into the oral cavity for treatment. Two months after his initial visit, the patient visited us complaining of pain in the same area. However, he had recently undergone plasmapheresis treatment in neurology to alleviate limited mouth opening and systemic myalgia, resulting in a pain-free mouth-opening distance of approximately 35 mm. During this temporary period in which he had no restriction in mouth opening, we performed tooth extraction and bridge restoration on the mandibular right first molar and created an oral appliance for sleep bruxism.

**Conclusions:**

Plasmapheresis therapy transiently reduced trismus, rendering dental interventions feasible, albeit temporarily. This case report underscores the importance of close collaboration between neurologists and dentists who encounter similar cases while furnishing valuable insights to inform dental treatment planning.

## Background

Isaacs’ syndrome is an autoimmune disorder characterized by spontaneous and/or continuous muscle fiber activity that results from peripheral nerve hyperexcitability (PNH) [1]. Clinical symptoms include muscle cramps, fasciculations, myokymia (i.e., undulating movements of the skin surface), and pseudomyotonia (i.e., delayed relaxation after contraction) [2]. Orphanet reported that although the prevalence of Isaacs’ syndrome remains unknown, there were approximately 100–200 reported cases across 41 countries by 2013 [3]. Initial treatment is symptomatic; however, immune therapy is often required and can be beneficial [2]. In particular, many investigators have noted a response to plasmapheresis treatment [4, 5].

Although generalized body aches, muscle stiffness, and excessive sweating are common symptoms reported in many case reports about Isaacs’ syndrome [4, 6, 7], there are very few reports of symptoms related to the oral condition. Paliwal reported a case in which myokymic discharges in the masseter muscle were recognized by electromyography in a patient with Isaacs’ syndrome with trismus. To the best of our knowledge [8], there have been no reports regarding dental treatment and oral management in patients with trismus.

## Case presentation

A 36-year-old male patient was referred to the Geriatric Dentistry and Perioperative Oral Care Center at Kyushu University Hospital (Fukuoka, Japan) with a primary complaint of swelling and pain in the gingiva surrounding his right lower first molar. Six years prior, he experienced an onset of rigidity in his masticatory musculature during breakfast, concomitant with cramping in both lower extremities. As these symptoms gradually worsened, he underwent various tests at the neurology department, resulting in a provisional diagnosis of Isaacs’ syndrome. He visited a dentist near his home 2 weeks before he presented to us, when he started noticing oral symptoms, and he was diagnosed with chronic apical periodontitis in his right lower first molar. Nevertheless, because of the underlying condition of Isaacs’ syndrome, he was advised against treatment or medications. During the primary care dentist’s visit, the patient was not treated for intraoral pain because of limited mouth opening. Therefore, the primary care dentist did not provide information regarding mouth opening; however, it appeared that the patient could slightly open his mouth. Persistent discomfort prompted the patient to seek guidance from his primary neurologist, ultimately resulting in referral to our department for further evaluation and management.

**Fig. 1:**
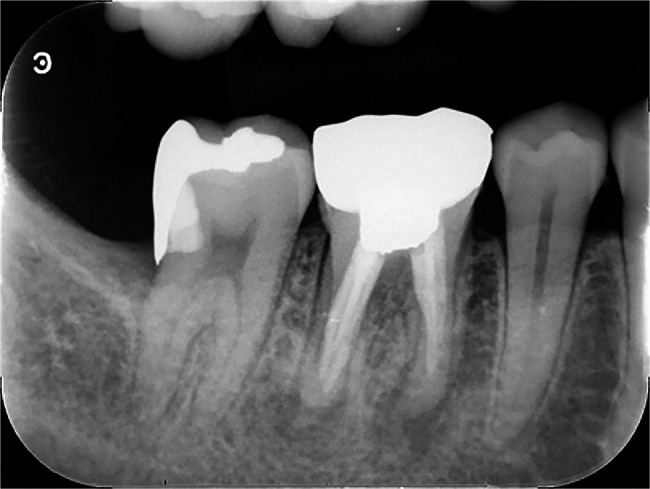
Images of the radiologic examination of the mandibular right first molar at the initial consultation

He arrived at our department in a motorized wheelchair, accompanied by his spouse. Transitioning to the dental chair and maintaining a horizontal position were achievable before the dental procedures. The patient’s painless mouth-opening distance (between incisors) was 20 mm. His facial appearance was symmetrical on both sides. A full cast crown was fitted to the mandibular right first molar, which had already undergone root canal treatment. A fistula was found in the periapical gingiva, and pain on percussion was detected. We observed diffuse X-ray translucency around the apical portion of the distal and mesial roots (Fig. 1). The main blood test results were as follows: white blood cell (WBC), 5.41 × 10^3^/µL; neutrophils, 60.6%; C-reactive protein, 0.09 (mg/dL).

Five months before visiting our department, the patient was examined by the Department of Oral and Maxillofacial Surgery at our hospital, where temporomandibular joint disorders were ruled out. At the time of diagnosis, the occlusion was normal with adequate coverage, the mandible deviated by 1 mm to the left, and a clicking sound was noted in the right mandible. Slight flattening of the condylar heads was observed in the mandible, although yet there was no compromise in mobility. A tingling sensation of paresthesia was noted near the posterior edges of the bilateral mandibular angles. No muscle pain was noted in the masseter, temporalis, or sternocleidomastoid muscles. Moreover, a hard end feel and soft end feel were not observed.

Based on these findings, a diagnosis of chronic apical periodontitis was established. We deemed that performing root canal treatment or tooth extraction would be challenging given the constrained jaw opening associated with Isaacs’ syndrome. Consequently, we elucidated to the patient the infeasibility of immediate tooth extraction or re-endodontic treatment under the current circumstances and proposed relieving symptoms through oral medication as an alternative approach. To consider potential drug interactions, we coordinated with the patient’s neurologist. Following a collaborative diagnosis, a regimen of amoxicillin capsules at a dosage of 750 mg was prescribed to be administered in three divided doses over a 3-day period. In addition, acetaminophen was prescribed for as-needed pain relief.

Two months after his initial consultation at our department, the patient returned with recurrent pain localized to the same tooth. Although a fistula was not evident in the periapical gingiva, pain on percussion was elicited. Notably, because the patient had undergone plasmapheresis therapy in the neurology department 1 week prior, with the goal of improving restricted mouth opening and systemic myalgia, the patient’s pain-free mouth-opening distance was 30 mm on the day of plasmapheresis and 35 mm at the dental visit. Given the patient’s expressed preference to mitigate the risk of pain recurrence during episodes of limited mouth opening, we deemed tooth extraction to be more appropriate than root canal therapy for his right lower first molar.

**Fig. 2:**
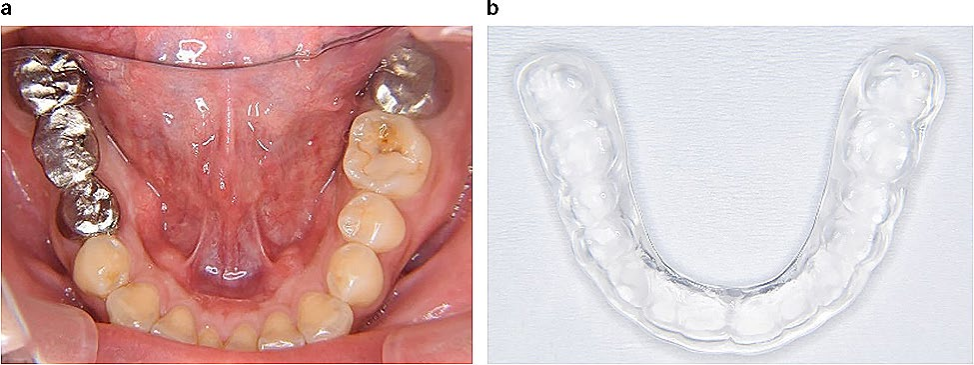
After the extraction of the mandibular right first molar and placement of a fixed prosthesis, an occlusal appliance was inserted for the prevention of teeth fracture

**Fig. 3:**
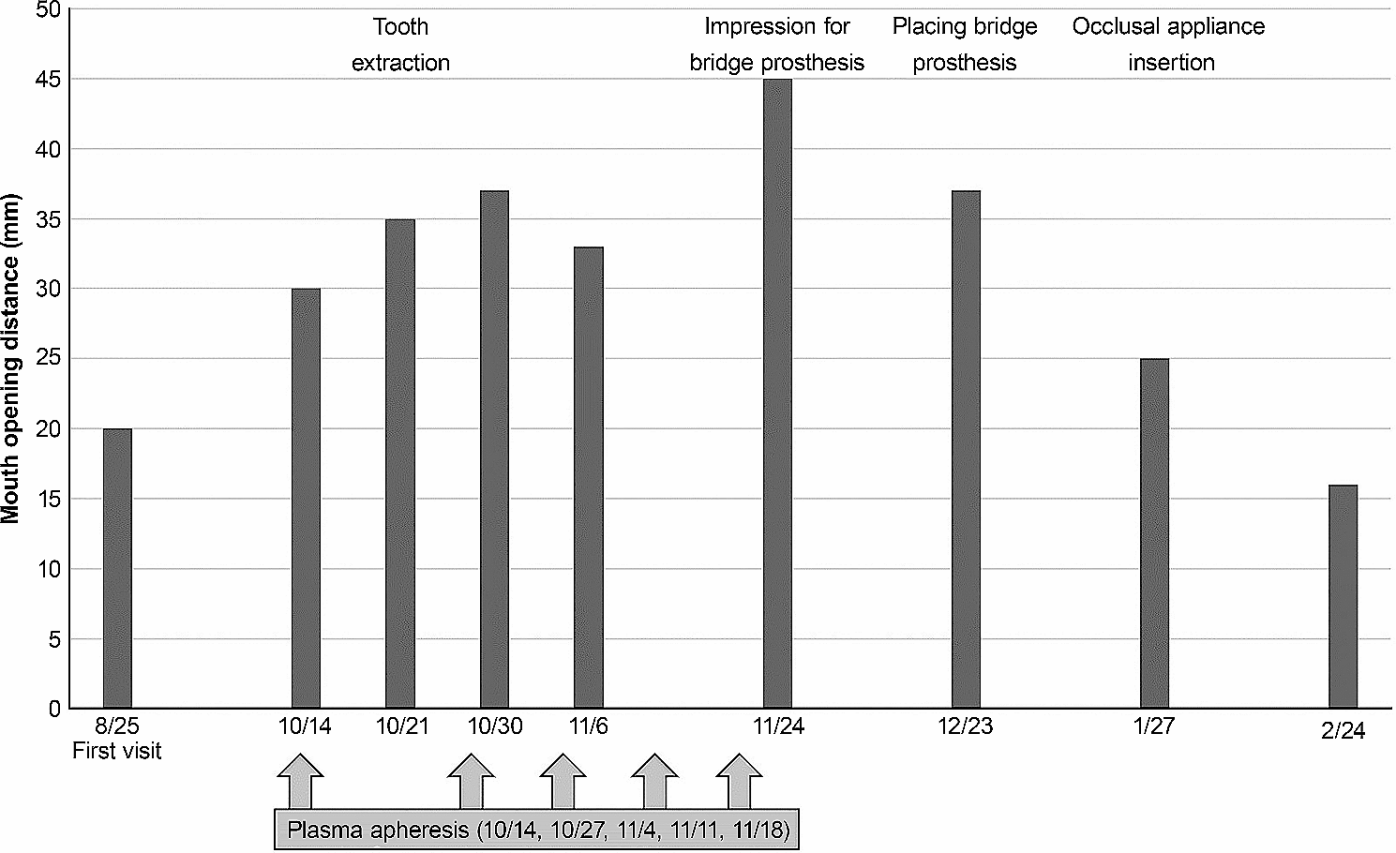
Plasma apheresis sessions were conducted on five occasions (October 14th, 27th, November 4th, 11th, and 18th), resulting in the amelioration of trismus symptoms for a period of approximately 3 months. Concurrently, interventions such as tooth extraction, bridge prosthesis placement, and occlusal appliance insertion were undertaken

Following the extraction procedure, we took dental impressions in order to fabricate a bridge prosthesis. Concurrent with plasmapheresis therapy, we initiated the fabrication of a bridge prosthesis, and an oral appliance for managing sleep bruxism was also fabricated (Fig. 2). One month after the completion of plasma exchange therapy, the patient’s mouth opening remained at a satisfactory level, exceeding 35 mm, which enabled the successful fitting of the bridge prosthesis. However, his mouth opening reduced to 25 mm after a 2-month interval. Nevertheless, we were able to design and implement an oral appliance to effectively manage nocturnal bruxism. Three months after plasma exchange therapy, his mouth opening decreased further to 16 mm (Fig. 3). The patient is currently undergoing maintenance for preventing the progression of periodontitis and for adjusting the oral appliance. Dentists provided rehabilitation instructions for the patient to regularly open his mouth fully upon waking up in the morning. Neurologists utilized pregabalin, baclofen, gabapentin, shakuyaku-kanzo-to, and loxoprofen for symptomatic management of myokymia, neuromyotonia, and related pain. The Japanese traditional herbal medicine Shakuyaku-kanzo-to has long been used for the treatment of muscle cramps that suddenly cause intense pain and those who get cramps in their legs during exercise or while sleeping [9].

## Discussion

In this patient with Isaacs’ syndrome, we performed tooth extraction and subsequent fabrication of both a prosthesis and night guard to prevent bruxism during sleep during a period when mouth opening increased after plasmapheresis therapy. The first-line treatment for chronic apical periodontitis is retreatment of an infected root canal. However, in this case, we selected tooth extraction after considering of the patient’s limited mouth opening caused by Isaacs’ syndrome and the limited period during which the mouth could be opened after plasmapheresis treatment. Furthermore, another factor in the treatment decision was our concern that the presence of an immune disorder could impair the success rate of infected root canal treatment [10]. In addition, the patient was aware that he clenched his teeth at night, so the use of a night guard was considered to prevent tooth fracture [11]. These treatments were performed during the approximately 3 months that the patient was able to open his mouth.

At the start of treatment, pharmacological interventions, for example, the administration of steroids and azathioprine or gabapentin, were attempted to alleviate symptoms; however, these were discontinued because of allergic reactions. Plasma exchange was not employed as a direct treatment for trismus. It was used as a symptomatic treatment for other symptoms of Isaacs’s syndrome, which also improves trismus. Although the masseter is smaller than the lower limb muscles, myokymia and neuromyotonia, which were present in other areas, may also affect the masseter. Consequently, once the masseter muscle contracted, it could not relax easily, leading to difficulty in opening the mouth. Plasma exchange was conducted for approximately 6 weeks, following which the patient could maintain a mouth opening distance of approximately 35 mm for 8–12 weeks. Subsequently, the ability to open the mouth gradually deteriorated. It was hypothesized that trismus, a symptom of Isaac’s syndrome, occurred following the contraction of the masseter followed by neuromyotonia. This occurs when patients clench their jaws day and night as a coping mechanism to endure widespread myalgia, a primary symptom of the syndrome. This neuromyotonia likely hinders muscle relaxation, resulting in difficulty opening the mouth. Plasma exchange was performed as symptomatic treatment, which alleviated, but did not fully resolve, the primary symptoms of Isaacs’ syndrome, such as neuromyotonia and myokymia. However, the neurologist in charge considered that even temporary improvements in systemic symptoms and easing of trismus for 8–12 weeks contribute significantly to enhancing the patient’s quality of life. Consequently, treatment with immunosuppression was not conducted, and plasma exchange was administered approximately every 6 months. Trismus improved with each plasmapheresis, indicating reproducibility.

Plasmapheresis is typically performed on a biannual basis, requiring an average hospital stay of approximately 5–6 weeks. During each hospitalization period, the procedure is performed every 7–10 days, requiring approximately 2–3 h per session, generally spanning five cycles. Therefore, developing a dental treatment plan that aligns with this schedule is recommended. On the other hand, we must also be careful about complications and adverse events caused by plasmapheresis therapy. Adverse events were mild in 2.4% (linked to vascular access in 54% and to the device- related in 7% of cases, besides hypotension and tingling), moderate in 3% (tingling, urticarial, hypotension, and nausea), and severe in 0.4% of treatments (hypotension and syncope, urticaria, chills or fever, arrhythmia or asystole, nausea, or vomiting) [12]. Thus, dentists should closely collaborate with the doctor in charge and understand the patient’s condition.

In Isaacs’ syndrome, neuromyotonia is characterized by an initial phase of progressive muscular rigidity, which eventually leads to extensive fasciculations across various muscles [1, 13]. This clinical progression serves as a hallmark of the disorder and highlights the underlying neuromuscular hyper excitability [1]. The symptom of limited mouth opening was considered to be one of the manifestations of neuromyotonia. In addition, a previous review reported that PNH syndromes such as Isaacs’ syndrome manifest clinically not only with neuromyotonia but also with chronic pain, muscle atrophy, and bulbar dysfunction [2]. Given the potential for not only restricted mouth opening but also possible symptoms such as decreased swallowing function, continuous clinical evaluations are deemed essential for comprehensive assessment and management. Moreover, the patient may occasionally clench his teeth to cope with the pain arising from muscle cramps during sleep, a phenomenon consistent with previous reports [1]. Given this behavior, we cannot discount the possibility of dental fractures or fissures leading to tooth pain [14, 15]. In addition, patients with masticatory muscle pain disorders exhibit high levels of the pain-related biomarker glutamine in their saliva and plasma [16]. Further studies are needed to examine the relationship between these biomarkers and clinical findings such as bruxism and trismus.

In this case, the patient had limited days available for dental visits, resulting in insufficient longitudinal data on mouth opening. Consequently, the patient had difficulty ruling out influences other than plasmapheresis. Thus, in similar cases, data on mouth opening must be recorded after plasma exchange.

We have been conducting follow-up observations for 3 years and 9 months since the initial consultation. The most crucial aspect is to understand the effectiveness and importance of self-care. When there is a reduction in mouth opening, active treatment, such as treatment of dental caries or tooth canal treatment is not feasible; therefore, maintenance is scheduled to coincide with visits to the Department of Neurology. Moreover, plasma exchange is costly and requires hospitalization, which can be burdensome. Consequently, plasma exchange was not conducted for the primary purpose of facilitating dental treatment. As trismus was present, there were periods when active dental treatment was challenging; therefore, we stressed the importance of daily effective brushing. In addition, numerous tooth cracks have been observed, prompting us to recommend the use of oral appliances to prevent tooth fractures, and we continue to monitor changes in occlusal relationships.

## Conclusions

Immediately after undergoing plasmapheresis treatment, during the brief period of unobstructed mouth opening, our patient with Isaacs’ syndrome was opportunistically administered intensive dental treatment. We suggest a close cooperation between physicians and dentists who may encounter similar cases.

## Data Availability

Data is provided within the manuscript.

## Abbreviations

PNH: Peripheral Nerve Hyperexcitability
